# Price per unit: the cost of alcohol-related admissions to a regional ICU

**DOI:** 10.1186/cc13263

**Published:** 2014-03-17

**Authors:** R Matthews, A Revill

**Affiliations:** 1 Plymouth Hospitals NHS Trust, Plymouth, UK

## Introduction

A number of studies have demonstrated the significant and increasing morbidity and mortality associated with alcohol-related disease in the UK, and the corresponding impact this has on critical care services [1]. This study was designed to similarly evaluate the current burden of alcohol-related disease on admission rates to a regional ICU, but also to calculate the financial costs of such admissions.

## Methods

Data-entry fields derived from recommendations in a public heath publication [2] were introduced to the local electronic records system to enable prospective data collection for all admissions that were either wholly or partially attributable to alcohol consumption. Using locally defined values for the cost per day of an admission to the ICU, depending on the maximum level of organ support required during the admission, it was possible to calculate the total expense incurred by the unit for each alcohol-related admission.

## Results

**Figure 1:**
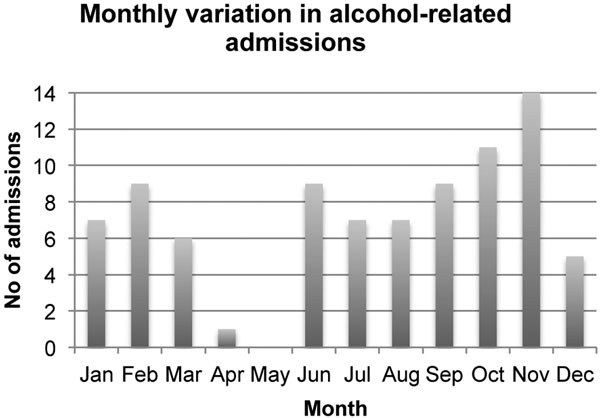


In 1 year from December 2012 to November 2013 inclusive, the ICU recorded 84 alcohol-related admissions, accounting for approximately 9% of annual unplanned admissions. With an average length of stay (5.8 days) similar to that of all other unplanned admissions, this totalled 534 ICU bed-days. A total of 86% of patients with alcohol-related conditions were male with an average age of 46.4 years (range 15 to 83 years), and the majority (42%) presented with chronic conditions partially attributable to alcohol consumption. The number of admissions per month varied from zero in May to a peak of 14 in November, with the majority (40%) of admissions occurring over the autumn months (Figure 1). Eighty-nine per cent of patients with alcohol-related conditions required support for at least two organ failures, which subsequently equated to an overall cost to the unit of £725,308 and 12% of an approximate £6 million annual budget.

## Conclusion

The results of this study support the hypothesis that alcohol-related disease contributes considerably to admissions to this ICU. Furthermore, they have shown that the financial impact was proportionally greater than the percentage number of admissions attributable to alcohol consumption, reflecting the high frequency of multiorgan failure in this patient cohort.
